# Endoscopic Submucosal Dissection of Deeply Invasive Colorectal Cancers Using the Pocket-Creation Method: Analysis of Vertical Margins

**DOI:** 10.3389/fgstr.2022.879615

**Published:** 2022-05-13

**Authors:** Takaaki Morikawa, Yoshikazu Hayashi, Hisashi Fukuda, Hiroaki Ishii, Tatsuma Nomura, Eriko Ikeda, Masafumi Kitamura, Yuka Kagaya, Masahiro Okada, Takahito Takezawa, Keijiro Sunada, Alan Kawarai Lefor, Noriyoshi Fukushima, Hironori Yamamoto

**Affiliations:** ^1^ Department of Medicine, Division of Gastroenterology, Jichi Medical University, Shimotsuke, Japan; ^2^ Department of Surgery, Jichi Medical University, Shimotsuke, Japan; ^3^ Department of Diagnostic Pathology, Jichi Medical University, Shimotsuke, Japan

**Keywords:** pocket-creation method, endoscopic submucosal dissection, ESD, colon, rectum

## Abstract

**Background and aims:**

The standard treatment for stage T1b colorectal cancers with 1,000µm or greater submucosal invasion is surgical resection. However, the risk of lymph node metastases is only 1-2% when excluding risk factors for metastases other than depth of submucosal invasion. The number of elderly patients with significant comorbidities is increasing with societal aging in Japan. Therefore, local endoscopic resection of T1b colorectal cancers needs more consideration in the future. We previously showed that the pocket-creation method (PCM) for endoscopic submucosal dissection (ESD) is useful regardless of the morphology, including large sessile tumors with submucosal fibrosis, or location of the colorectal tumor. However, some T1b colorectal cancers have pathologically positive margins even when using the PCM. We retrospectively investigated the causes of failure to achieve negative vertical margins.

**Methods:**

We retrospectively analyzed 953 colorectal tumors in 886 patients resected with the PCM. Finally, 65 pathological T1b colorectal cancers after *en bloc* resection were included in this study. ESD specimens and recorded procedure videos of T1b cancer resections with pathologically positive vertical margins were reviewed.

**Results:**

The 65 cancers were divided into positive vertical margin (VM+ group) and negative vertical margin (VM- group) groups with 10 [10/65 (15%)] and 55 [55/65 (85%)] patients in each group, respectively. There was a significant difference in the rate of submucosal fibrosis (P=0.012) and dissection speed (P=0.044). There were no significant differences between the two groups in other regards. When verifying 8/10 available videos in the VM+ group, endoscopic technical factors led to positive vertical margins in five patients, and essential pathological factors of ESD led to positive vertical margins in the other three. Six of these eight patients underwent additional surgical resection. No residual tumor was identified in six T1b cancers. None of these six resected specimens contained lymph node metastases on pathological examination.

**Conclusion:**

The PCM resulted in a high rate of negative-vertical-margin resections. The PCM resulted in complete resection of T1b cancers when examining additional surgical specimens. ESD using the PCM is a viable option for the endoscopic treatment of T1b colorectal cancers.

## Introduction

The standard treatment for stage T1b colorectal cancers with 1,000µm or greater submucosal invasion is surgical resection because the risk of lymph node metastases is 12.5% according to the Japanese guideline (1). However, the risk of lymph node metastases is only 1-2% when excluding other risk factors for metastatic disease such as lympho-vascular invasion, budding, and poorly differentiated cancer (2, 3). Other reports described that the risk of lymph node metastases is approximately 0.8-1.3% excluding other risk factors when the pathologist determined the tumor grade based on the most unfavorable area, even if it was a small region (4, 5). Therefore, expanded indications for endoscopic treatment have been discussed (2, 6, 7). Together with the increase in the number of elderly patients with comorbidities, local resection with/without chemoradiation is considered an alternative to surgical resection including lymph node resection for stage T1b rectal cancers in clinical trials (JCOG1612). Endoscopic local resection of stage T1b colorectal cancers will be required more frequently in the future. As a requisite for endoscopic local resections, pathologically negative vertical margins should be achieved in endoscopically resected specimens.

The pocket-creation method (PCM) was developed as a technique for colorectal endoscopic submucosal dissection (ESD) (8). We have shown that the PCM is useful regardless of the morphology, including large sessile tumors with submucosal fibrosis, or location of the colorectal tumor and is also more effective than conventional colorectal ESD (9–11). Although large colorectal sessile tumors commonly have severe submucosal fibrosis, we showed that the PCM facilitates ESD of tumors with a higher rate of negative vertical margins (12, 13). This is because the submucosa is dissected just above the muscularis, since the small-caliber-tip transparent hood (ST hood, Fujifilm, Tokyo, Japan) stretches the submucosal tissue allowing one to visually identify the surface of the muscularis during the PCM of ESD. Although the PCM is the most suitable way to perform endoscopic local resection of stage T1b cancers, there have been some T1b colorectal cancers with pathologically positive vertical margins even using the PCM. Therefore, we retrospectively investigated the causes of failure to achieve negative vertical margins.

## Materials and Methods

### Study Design and Participants

**Figure 1:**
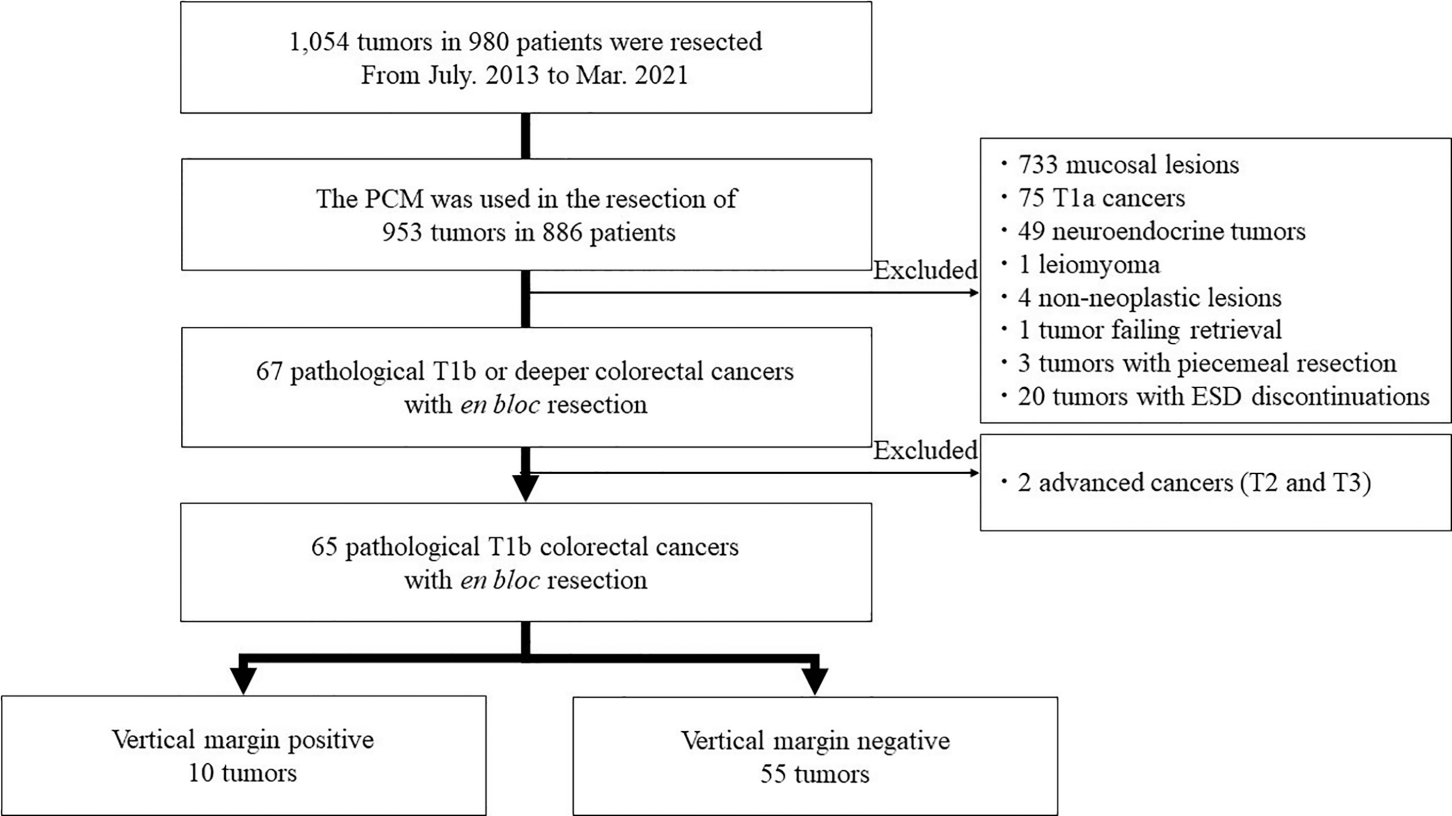
Flow chart for the inclusion of lesions in this study.

This is a single-center retrospective study. All data was extracted from the ESD database of Jichi Medical University, reflecting patients cared for between July 2013 and March 2021 (**Figure 1**). A total of 1,054 tumors in 980 patients were resected during this period. The PCM was used for the resection of 953 tumors in 886 patients. Of these, 733 mucosal lesions, 75 T1a cancers with submucosal invasion shallower than 1,000µm,49 neuroendocrine tumors, four non-neoplastic lesions, one tumor failing retrieval, and one leiomyoma originating from the muscularis were excluded. Three tumors with failure of *en bloc* resection and 20 tumors with discontinuation of ESD were excluded. The purpose of this study was to assess the utility of ESD with the PCM for pathologically diagnosed deep submucosal invasive cancers by analyzing their vertical margins. Therefore, we excluded patients who had a piecemeal resection or for whom ESD was discontinued because reliable pathologic evaluation was impossible. Two tumors with T2 and T3 were excluded because this study is focused on the technical aspects of the PCM for T1b cancers. Finally, 65 pathological stage T1b colorectal cancers obtained by *en bloc* resection were analyzed in this study. Written informed consent for undergoing ESD was obtained from all patients. Informed consent for inclusion in this study was not required because it is limited to a retrospective, anonymous data analysis. This study complied with the Declaration of Helsinki and the current code of ethics and was approved by the Ethics Committee of Jichi Medical University (No. 20-103).

### Data Collection

Gender, age, tumor location, tumor morphology, endoscopic depth prediction, resected tumor diameter, specimen diameter, dissection time, dissection speed, degree of submucosal fibrosis, histopathology, lympho-vascular invasion, evaluation of horizontal and vertical margins (negative or positive), and occurrence of complications (perforation, delayed bleeding) were extracted from the ESD database. Tumor locations were classified as in the colon or rectum. The colon was defined from the cecum to the rectosigmoid segment. Dissection time was defined as the time from the mucosal incision to the end of the resection. Dissection speed was defined as specimen area/dissection time (mm^2^/min) (14). Submucosal fibrosis was classified as F0, F1, or F2 according to previous studies (15). Perforation was defined as a defect in the muscularis propria and serosa that allowed flow between the inside and outside of the intestinal lumen. Delayed bleeding was defined as overt bleeding within 14 days after ESD requiring endoscopic hemostasis or causing hemoglobin to drop >2g/dl. Post-ESD electrocoagulation syndrome was defined as localized abdominal tenderness and fever (≥37.6 °C) or an inflammatory reaction [leukocytosis (≥10,000 cells/μL) or increased CRP (≥0.5)] without definitive evidence of perforation, which occurred 6 h or later after colorectal ESD according to criteria from a previous study (16). When patients underwent additional surgical resection, the histopathology of the resected specimen was also recorded.

### Clinical Indications for ESD

Prediction of depth of invasion before ESD is performed by magnification and/or image-enhanced endoscopy and/or endoscopic ultrasound using miniature probe. The indications for colorectal ESD included the following criteria as described in our previous report (11): (1) magnifying and/or image-enhanced endoscopy did not suggest deep submucosal invasion of 1000 µm and (2) lesions difficult to resect with EMR in an *en bloc* fashion. Difficulty related to lesion size, location, or shape is not an exclusion criterion in our institution. In addition, after obtaining careful informed consent from the patient, ESD even of lesions with suspected 1000µm or deeper submucosal invasion may be performed for relative indications and/or a total excisional biopsy.

### Equipment and Devices Used for ESD

We used the following devices for ESD:

-Therapeutic endoscopes with a narrow diameter, an accessory channel of 3.2mm in diameter, and a water jet channel (EG-450RD5, EG-L580RD7, and EC-580RD/M; Fujifilm, Tokyo, Japan),
-A small-caliber-tip transparent (ST) hood (DH-15GR, DH-28GR, and DH-33GR; Fujifilm),
-Sodium hyaluronate 0.4% (MucoUp; Boston scientific Co., Tokyo, Japan) with 0.002-0.004% indigo carmine and 0.001% epinephrine as the submucosal injection solution,
- Needle-type knives such as the DualKnife (KD-650Q; Olympus, Tokyo, Japan), DualKnife J (KD-655Q; Olympus), Flushknife BT (DK2618JB-15-; Fujifilm), or Flushknife BT-S (DK2620J-B15S-; Fujifilm),
-Hot hemostatic forceps (HOYA Corporation, Tokyo, Japan) to control intraprocedural bleeding,
-VIO-300D (ERBE Elektromedizin GmbH, Tuebingen, Germany) with Endocut I (effect 1, duration 4, Interval 1) for mucosal incision, Swift coagulation (effect 4, 25-30W) for submucosal dissection, soft coagulation (effect 4, 80W) for hemostasis as diathermy, and
-When endoscopic maneuvering was unstable, balloon-assisted endoscopes (EN-450BI5 and EI-580BT; Fujifilm) with an overtube (TS-13101, Fujifilm) were used (17).

### The Pocket-Creation Method

**Figure 2:**
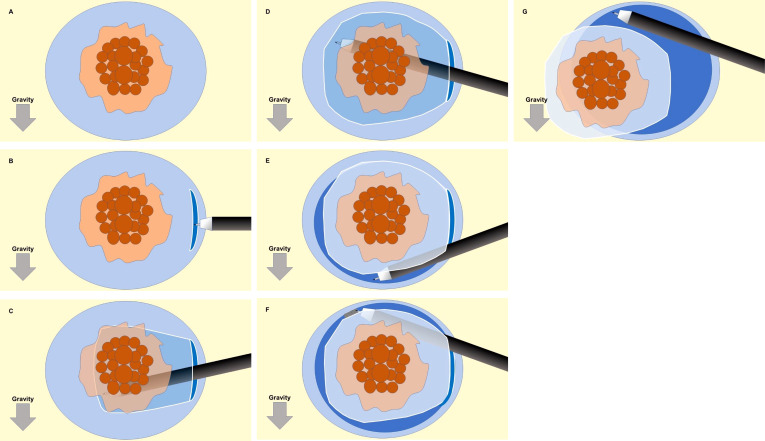
Schematic illustration of the sequence for the pocket-creation method of endoscopic submucosal dissection (ESD). **(A)** A circumferential submucosal injection is performed around the entire tumor. **(B)** An initial mucosal incision approximately 15 mm from the distal edge of the tumor was made. The width of the incision should be less than 2cm. **(C, D)** The submucosal layer was dissected to create a submucosal pocket under the tumor. **(E)** After dissecting the submucosa below the tumor, the pocket was opened in a step-by-step manner, with repetition of the mucosal incision and subsequent submucosal dissection, up to the proximal margin of the tumor. **(F, G)** The upper remaining area was opened to complete the ESD.

First, a circumferential submucosal injection was performed around the entire tumor. An initial mucosal incision approximately 15 mm from the distal edge of the tumor was made. The width of the incision should be less than 2cm. The submucosal layer was dissected to create a submucosal pocket under the tumor. In the pocket, both traction and countertraction produced by the ST hood efficiently stretched the submucosal tissue, which allowed identification of the surface of the muscularis and dissection of the submucosa just above the muscularis. After dissecting the submucosa below the tumor, the pocket was opened in a step-by-step manner, with repetition of the mucosal incision and subsequent submucosal dissection, up to the proximal margin of the tumor. Finally, the upper remaining area was opened to complete the ESD (**Figure 2**).

### Analysis of Positive Vertical-Margin Stage T1b Cancers

Specimens and recorded videos for ESD procedures of stage T1b cancers with pathologically positive vertical margins were reviewed by three physicians (T.M, H.F, Y.H), two board-certified fellows and one board-certified trainer of the Japan Gastroenterological Endoscopy Society, to classify the causes of positive margins as either endoscopic technical factors or essential pathological factors that do not depend on ESD skill. Technical factors were defined as a shallow dissection level leaving submucosa above the muscularis and/or an accidental cauterization on the cancer. Essential pathological factors were defined as the submucosa between the cancer and the muscularis thinner than a knife-tip diameter (DualKnife; 0.65 mm, Flushknife BT and BT-S; 0.90 mm).

### Statistical Analysis

The Mann-Whitney U-test was used to analyze continuous or ordinal variables for comparison between patient groups. The chi-square test or Fisher’s exact test was used for categorical data. In comparisons of submucosal fibrosis, *P* values were interpreted after Bonferroni’s correction for multiple comparisons. A significance level with a p-value of 0.05 or less was considered significant. EZR (Saitama Medical Center, Jichi Medical University, Saitama, Japan) was used for all statistical analyses (18).

## Results

### Baseline Characteristics

Sixty-five patients with stage T1b colorectal cancers who underwent *en bloc* resection using the PCM (**Table 1**) were analyzed. Patients were divided into two groups, those whose lesions had a positive vertical margin (VM+ group) and those with a negative vertical margin (VM- group). The number of patients in each group were 10 [10/65 (15%)] and 55 [55/65 (85%)], respectively. There were no significant differences between the two groups with regard to patient characteristics such as gender, age, tumor morphology, tumor location, and endoscopic depth prediction.

**Table 1:** Clinical characteristics of patients and treatment results for pathological stage T1b cancers.

	VM+(n=10)	VM-(n=55)	P value
Gender (male)	5 (50%)	30 (55%)	1
Age, y/o (range)	68.5(56-83)	68.0 (43-89)	0.434
Location			
Colon/Rectum	7 (70%)/3 (30%)	27 (49%)/28 (51%)	0.309
Tumor morphology			
Ip/Is/IIa	0/5/5	1/32/22	0.772
Clinical diagnosis			
cTis-cT1a/cT1b	7 (70%)/3(30%)	49 (89%)/6(11%)	0.135
Resected tumor diameter (mm)	23.0 (11.0-40.0)	26.0 (12.0-78.0)	0.291
Specimen diameter (mm)	37.5 (17.0-51.0)	40.0 (22.0-95.0)	0.295
Dissection time (min)	65.5 (31-152)	63.0 (21-248)	0.682
Dissection speed (mm^2^/min)	12.4 (4.3-26.6)	16.8 (7.1-53.6)	0.044
Submucosal fibrosis			
F0	0 (0%)	20 (36%)	
F1	4 (40%)	23 (42%)	0.012*
F2	6 (60%)	12 (22%)	
Pathological findings			
Histopathology (tub1/tub2/pap/muc)	6 (60%)/3 (30%)/0/1 (10%)	37 (67%)/16 (29%)/2 (4%)/0	0.263
Horizontal margin positive	1 (10%)	3 (6%)	0.496
Lymphatic vessel invasion	3 (30%)	20 (36%)	1
Venous invasion	5 (50%)	17 (31%)	0.287
Complication			
Perforation	0	1 (2%)	1
Delayed bleeding	0	2 (4%)	1
Post-ESD electrocoagulation syndrome	0	1(2%)	1

VM+, positive vertical margin; VM-, negative vertical margin; tub1, well-differentiated tubular adenocarcinoma; tub2, moderate-differentiated tubular adenocarcinoma; pap, papillary adenocarcinoma; muc, mucinous adenocarcinoma.

*Significant difference between F0 and F2 after Bonferroni’s correction.

### Therapeutic Outcomes

There was a significant difference between the two groups regarding submucosal fibrosis graded F0 and F2 determined using Fisher’s exact test with Bonferroni’s correction (p=0.012). The dissection speed was 12.4 and 16.8 mm^2^/min in the VM- and VM+ groups, respectively, and there was a significant difference between the two groups (P=0.044). However, there were no significant differences between the two groups in resected tumor diameter, specimen diameter, dissection time, histopathologic type, lymphatic invasion, venous invasion, or incidence of complications (**Table 1**).

### Analysis of Vertical-Margin-Positive Resections

**Figure 3:**
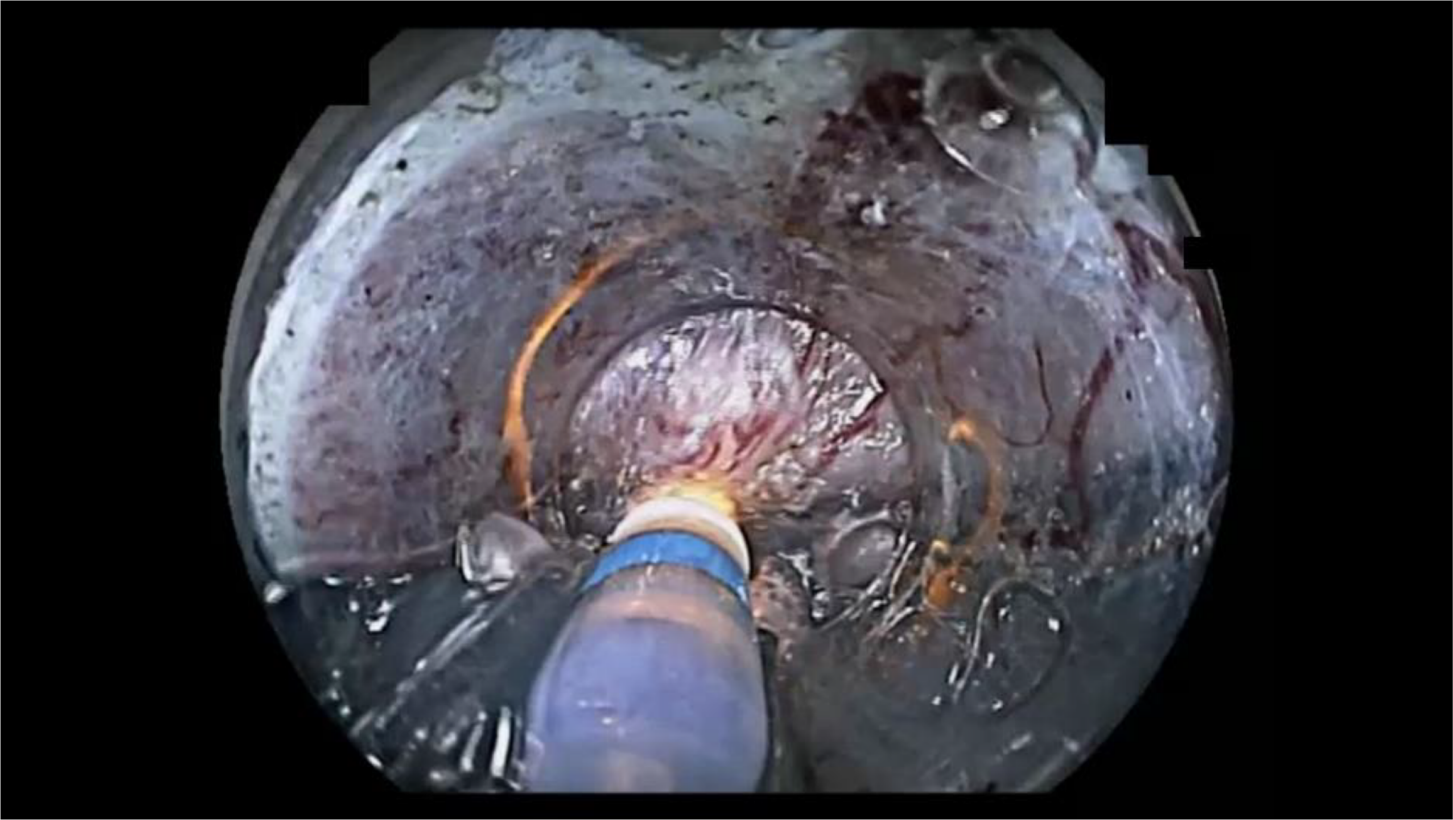
An example of endoscopic submucosal dissection (ESD) factors identified as “endoscopist’s technical factors” in patient 2 (**Table 2**). The ESD knife incidentally cauterized the cancer because the submucosa was not stretched using the ST-hood tip.

**Figure 4:**
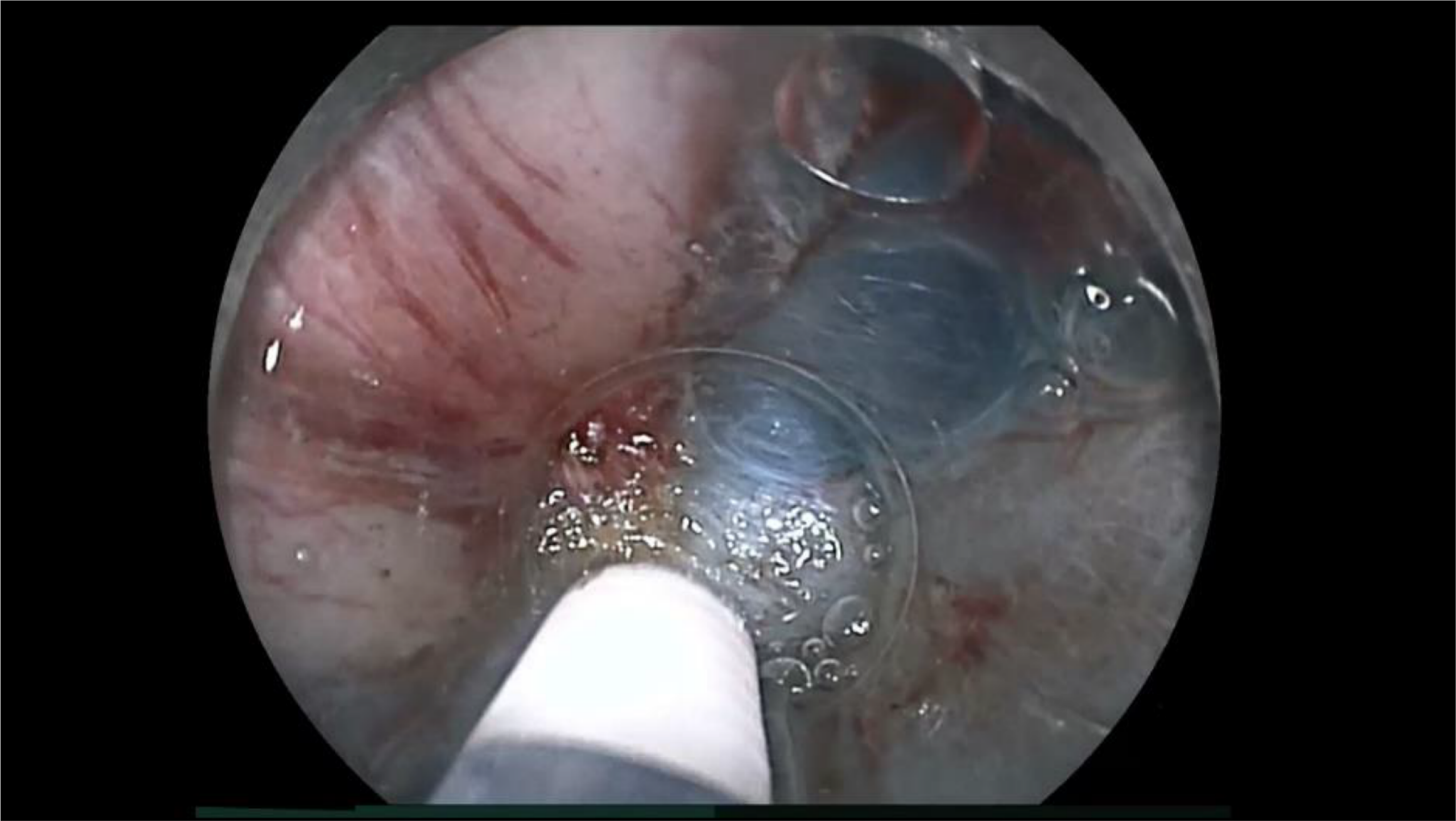
An example of endoscopic submucosal dissection (ESD) factors identified as the “essential pathological factors” in patient 10 (**Table 2**). Although the submucosa was stretched by the ST-hood tip as much as possible, fibrotic submucosa between the cancer and the muscularis was still thinner than the tip of ESD knife. Therefore, the cancer could not be resected without its incidental cauterization.

ESD procedure videos for lesions in the VM+ group were reviewed to identify the causes of positive vertical margins (**Table 2**). Two of 10 patients did not have videos available and the remaining ten ESD videos were reviewed. Review showed that technical factors associated with the endoscopist resulted in positive vertical margins in five patients (**Figure 3**). It was judged that essential pathological factors resulted in positive vertical margins in the other three patients (**Figure 4**). In addition, 6/8 patients underwent additional surgical resection. Pathologic evaluation of the resected specimens did not show any lymph node metastases in all six patients.

**Table 2:** Characteristics of vertical-margin-positive cases.

Patient	Gender	Age	Location	Morphology	Submucosal fibrosis	Pathology	SM invasion distance (μm)	Budding	Ly	V	Surgical pathology	Lymph node metastasis	Video validation
1	F	61	A	Is	F2	tub2	>3000	X	1	1	No remains	None	Essential pathological factors
2	M	64	A	IIa	F1	tub1	>1000	X	0	1	No remains	None	Technical factors
3	M	78	Rb	IIa	F2	tub1	>4000	X	0	1	–	–	–
4	F	70	S	IIa	F2	tub1	>1000	X	0	1	No remains	None	Technical factors
5	M	67	RS	IIa	F2	tub2	>2000	X	0	0	No remains	None	–
6	F	56	S	Is	F1	tub1	>4000	2	1	0	No remains	None	Technical factors
7	M	80	T	IIa	F1	tub2	>2500	2	1	1	–	–	Technical factors
8	M	83	Rb	Is	F1	tub1	>7000	X	0	0	–	–	Technical factors
9	F	66	S	Is	F2	muc	>8600	1	0	0	No remains	None	Essential pathological factors
10	F	75	A	Is	F2	tub1	>6000	1	0	0	No remains	None	Essential pathological factors

A, ascending colon; T, transverse colon; S, sigmoid colon; RS, rectosigmoid; Ra, upper rectum; Rb, lower rectum.

## Discussion

Patients with colorectal T1b cancers have a risk for developing lymph node metastases. As an indication in the guidelines, additional surgery should be performed even if an endoscopic R0 resection was successful. However, rates of developing lymph node metastases differ among T1b cancers depending on the presence of risk factors for metastases. In addition, the level of tolerance for additional surgery may vary according to the patient’s age, comorbidities, and site of lesions. Therefore, it is acceptable to choose between endoscopic treatment as a relative indication for ESD or additional surgery for T1b cancers, depending on reliability of the assessment of risk factors for metastases based on an accurate pathological diagnosis and individual risk tolerance. The PCM under visual dissection facilitates a reliable R0 resection. The PCM allows for resection with no residual lesions and a highly reliable estimation of the risk of developing metastases through accurate pathological evaluation.

**Figure 5:**
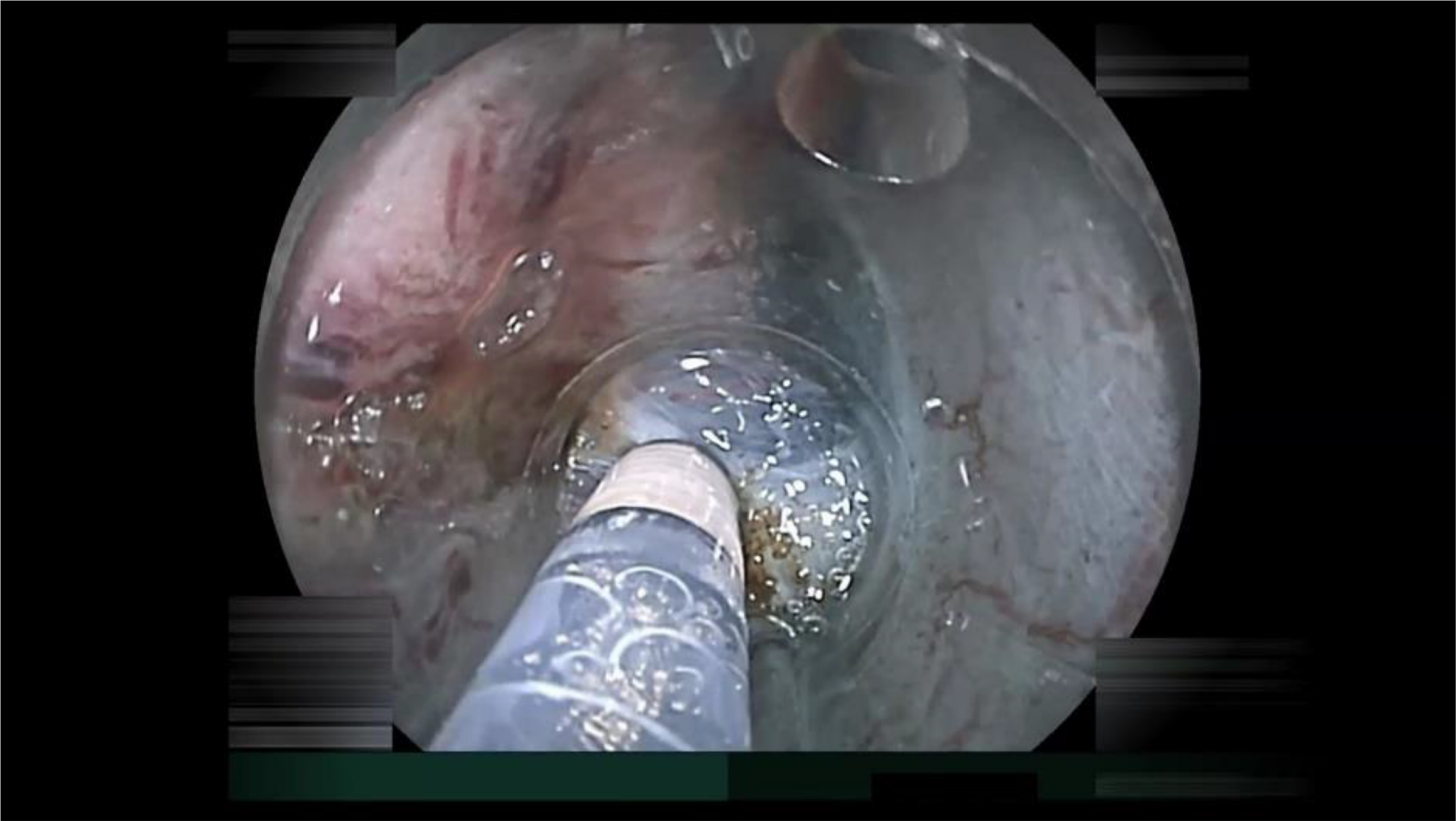
The small-caliber-tip transparent hood inserted into the limited space of the submucosal pocket naturally produces both traction and countertraction to stretch the submucosa. The stretched submucosa can be cut precisely with identification of the deepest tip of an invading cancer under direct vision.

The PCM for ESD facilitates a high rate of negative vertical margins in the resected specimens (85%) despite being used to resect stage T1b colorectal cancers. Severe submucosal fibrosis (F2) was significant among lesions in the VM+ group (P=0.012). The dissection speed was significantly slower in the VM+ group (P=0.044). Extremely thin submucosa with severe fibrosis under the lesion would lead to cauterization of the vertical edge of the cancer during ESD, which would result in a positive vertical margin. Furthermore, the dissection speed was also reduced because careful manipulation was required to dissect the submucosal layer which was narrowed by severe fibrosis. Similar to the results of the present study, previous reports have shown that dissection speed is reduced for ESD of colorectal lesions with severe fibrosis (19, 20). However, when examining surgical specimens, no residual tumor nor lymph node metastasis were detected pathologically for all stage T1b cancer cases required additional surgery. Therefore, this study suggests that even stage T1b cancers can be completely resected with the PCM for ESD. The *en bloc* resection rate of colorectal ESD was reportedly 94.5% in a Japanese multicenter study (21). Asayama et al., reported in a study from a single Japanese center that the *en bloc* resection rate and R0 resection rate of ESD for stage T1 cancers are 100% (37/37) and 91.9% (34/37), respectively. All three stage T1 cancers in that study without R0 resection had deep submucosal invasion. Two of them had severe submucosal fibrosis. The other one contained poorly differentiated cancer at the front of the invasion (22). Watanabe et al. reported that there was no difference in *en bloc* resection rate between stage T1a and T1b cancers (98.7 vs. 100%). However, they showed the R0 resection rate was significantly higher in stage T1a cancers than stage T1b cancers (97.4 vs. 64.7%). Their rate of positive vertical margins was significantly lower for stage T1a cancers than T1b cancers (0% vs. 35.3%) (23). Although the present study shows that cancers in the VM+ group had a significantly higher rate of severe fibrosis than those in the VM- group (P=0.012) as in previous reports, the rate of pathologically positive vertical margins with stage pT1b cancers (15% [10/65]) was lower than in the previous reports. This result suggests that the PCM is a viable option for ESD of stage T1b colorectal cancer. The reasons why the PCM achieves a high rate of negative vertical margins even in the resection of stage T1b cancers is that this technique keeps the submucosa thick because of a minimal mucosal incision and stretching of the thin and/or fibrotic submucosal tissue using the ST hood. The PCM can prevent leakage of submucosally injected solution because the mucosal incision is minimal, which keeps the submucosa thickened for a long time. The long-lasting thick submucosa also allows the endoscopist to easily identify the submucosa between the cancer and the muscularis. Insertion of the conical-shaped ST hood into the limited space of the submucosal pocket naturally produces both traction and countertraction, stretching the submucosa. This allows the stretched submucosa to be cut precisely with identification of the deepest tip of an invading cancer under direct vision (**Figure 5**). In general, when tightly stretching even soft objects such as cloth or paper, they can be cut efficiently with scissors without excessive physical energy. Similarly, the stretched submucosa can be cut with minimal thermal energy, which prevents the vertical margin from being damaged by burning or scarring.

However, some stage T1b cancers cannot be resected with negative vertical margins. Even if the ST hood stretches the thin and/or fibrotic submucosa under the invading cancer, the stretching will be limited. When the stretched submucosa is thinner than the tip of the ESD knife used, it is inevitable to cauterize the front of the cancer. To completely overcome this problem, we must remove the cancer together with the inner layer of circular muscle. The technique has been established similar to anal endoscopic myotomy with an internal myotomy in the rectum (24). Recently, Fukuda et al. reported complete ESD of a stage T1b cecal cancer with muscle retraction after clipping the retracted muscle with a reopenable clip to prevent immediate perforation (25). However, both procedures can only be performed by skilled endoscopists in limited situations. Although Guillaumot, et al. reported the feasibility of endoscopic full-thickness resection (26), there are still issues that need to be resolved, such as the risk of perforation with the development of peritonitis and intraperitoneal dissemination of the tumor.

When focusing on depth prediction prior to ESD for pathological T1b cancers, 56 (86%) and nine (14%) of them were diagnosed as clinical stages Tis-T1a and T1b, respectively (**Table 1**). Therefore, 86% of the pathological T1b cancers were understaged. Meanwhile, 14% of the pathological stage T1b cancers were intentionally treated with ESD using the PCM for a relative indication. The PCM allowed 88% (49/56) of pathological T1b cancers which were understaged as clinical stage Tis-T1a and 67% (6/9) of correctly diagnosed T1b cancers to be resected with pathologically negative vertical margins (P=0.135). We analyzed only pathological stage T1b cancers as research targets in this study. Therefore, it can be said that these 65 results reflect characteristics of relative-indications for ESD. This study indicates that ESD with the PCM may be a useful therapeutic technique even for pathological stage T1b cancers as a relative indication. However, in actual clinical practice, the indications for ESD must be determined based on the clinical diagnosis, including endoscopic prediction of pathological depth. We may underestimate or overestimate cancer depth prior to ESD in practice. Even if the cancer is limited to pathological stage Tis-T1a, ESD may be technically challenging due to severe submucosal fibrosis, etc. It is essential to determine a therapeutic plan with an understanding of the limitations of preoperative clinical diagnosis and the technical limitations of endoscopic treatment. Therefore, the PCM is a useful technique for total excisional biopsy and relative indications for ESD for cancers suspected to have submucosal invasion because the PCM allows us to dissect the submucosa under direct visualization of the vertical margin despite their submucosal invasion depth.

This study suggests the following important considerations for the endoscopic treatment of T1b colorectal cancers. First, ESD of cancers accompanied by severe submucosal fibrosis should be performed by expert endoscopists to avoid pathologically positive vertical margins due to technical factors. In this study, there were significantly more instances of severe fibrosis in patients with positive vertical margins than in patients with negative vertical margins. Second, even if the vertical margin of a resected specimen is positive, the cancer might have been completely removed by ESD when the submucosa was divided under the cancer with visual identification of the front of cancer invasion during ESD using the PCM. The PCM for ESD also allows us to dissect the submucosa right above the muscularis by direct visual identification.

While understanding the limitations of ESD, we must make efforts to achieve negative vertical margins. Toward this goal, we believe that the PCM is optimal because the deepest tip of a cancer invading to the submucosa is identified under direct vision in the pocket and dissection proceeds under the tip precisely.

There are several limitations to this study. First, this is a single-center retrospective study. Second, there is a relatively small number of patients because colorectal ESD is formally indicated only for up to stage T1a cancers in Japan. It is necessary to continue to accumulate cases to validate these results. Third, there could be significant selection bias in the present study. We analyzed only pathological stage T1b cancers because this study focused on the technical aspects of ESD with the PCM for colorectal submucosal invasive cancers. Both piecemeal-resection lesions for which pathological margins cannot be assessed accurately and advanced cancers as revealed by additional surgical resection were excluded from this study. Fourth, long-term follow-up results are needed to evaluate the appropriateness of non-surgical follow-up after ESD for stage T1b lesions.

In conclusion, the PCM is a viable option for the endoscopic treatment of stage T1b colorectal cancers.

## Data Availability Statement

The original contributions presented in the study are included in the article/supplementary material. Further inquiries can be directed to the corresponding author.

## Ethics Statement

The studies involving human participants were reviewed and approved by Ethics Committee of Jichi Medical University (No. 20-103). Written informed consent for participation was not required for this study in accordance with the national legislation and the institutional requirements.

## Author Contributions

TM, YH, HF: conception and design, analysis and interpretation of the data, drafting of the article. HI, TN, EI, MK, MO, TT, KS, NF: analysis and interpretation of the data, critical revision of the article for important intellectual content. AL: drafting of the article, critical revision of the article for important intellectual content. HY: conception and design, analysis and interpretation of the data, drafting of the article, critical revision of the article for important intellectual content. All authors reviewed and approved the final manuscript and final responsibility for the decision to submit for publication.

## Conflict of Interest

HY has a patent for the ST hood produced by Fujifilm Corporation. The hood was used for ESD and shown in figures. He also has a consultant relationship with Fujifilm Corporation and has received honoraria, grants, and royalties from the company.

The remaining authors declare that the research was conducted in the absence of any commercial or financial relationships that could be construed as a potential conflict of interest.

## Publisher’s Note

All claims expressed in this article are solely those of the authors and do not necessarily represent those of their affiliated organizations, or those of the publisher, the editors and the reviewers. Any product that may be evaluated in this article, or claim that may be made by its manufacturer, is not guaranteed or endorsed by the publisher.
